# Safe spaces, valued voices, and altruistic intentions: a qualitative study examining factors influencing Black women experiencing infertility and their willingness to participate in medical research

**DOI:** 10.1016/j.xfre.2026.04.005

**Published:** 2026-04-20

**Authors:** Morine Cebert, Shoshana Aronowitz, Rose Onyeali, Leah Field, Lisa Lewis

**Affiliations:** a Rory Meyers College of Nursing, New York University, New York, New York; b School of Nursing, University of Pennsylvania, Philadelphia, Pennsylvania; c Perelman School of Medicine, University of Pennsylvania, Philadelphia, Pennsylvania; d Anne Spencer Daves College of Education, Health, and Human Sciences, Florida State University, Tallahassee, Florida; e School of Nursing, University of the Virgin Islands, St. Croix, Virgin Islands

**Keywords:** Infertility, health disparities, research, recruitment

## Abstract

**Objective:**

To explore factors influencing Black women experiencing infertility to willingly engage in a research study, with the aim of informing ethically responsive recruitment strategies.

**Design:**

A qualitative secondary data analysis using inductive thematic analysis of semistructured interview data.

**Subjects:**

A total of 13 Black women with self-reported infertility who previously participated in a reproductive health research study.

**Exposure:**

Participant experiences and perspectives regarding decision-making processes for participation in infertility-related research.

**Main Outcome Measures:**

Perceived motivators, facilitators, and contextual factors influencing participation in reproductive health research among Black women.

**Results:**

Three major themes emerged as key influences on participation: Safety—participants emphasized the importance of psychological and cultural safety and trust in the research process; Valued voices—participants viewed their contributions as meaningful opportunities to amplify the experiences of Black women in infertility research; and Altruistic intentions—many women expressed a commitment to helping others by advancing awareness and improving care for future patients.

**Conclusion:**

Black women’s decisions to participate in reproductive health research are guided by feelings of safety, voice, and altruism. Researchers aiming to improve diversity in infertility studies should center participants’ emotional safety, ensure cultural responsiveness, and explicitly communicate the value of participants’ contributions. These findings provide a practical framework for ethically enhancing recruitment and engagement of underrepresented groups in clinical research.

## Introduction

Incorporating diverse racial samples in research studies has been shown to increase ‟generalizability of research findings, equity in provision of health care, and accuracy of ethnicity-specific subgroup analyses” (1). However, recruitment of Black women into clinical research trials remains a significant challenge across many healthcare disciplines, reflecting broader patterns of exclusion in women’s health research, where Black women are consistently underrepresented in studies of gynecologic care, maternal health, oncology, and chronic disease (2, 3, 4). Mistrust and discriminatory practices are well-documented factors contributing to these challenges. Historical abuses such as the Tuskegee syphilis study, the nonconsensual use of Henrietta Lacks’ cells, and the legacy of gynecologic experimentation by J. Marion Sims continue to shape collective mistrust of medical research (5). Many researchers, especially in the healthcare field, continue to contend with the extensive mistrust Blacks experience with the medical system because of decades of systemic racism and unethical research practices (6, 7, 8). Further, discrimination in research priorities has also had lasting effects. In 1986, the National Institutes of Health (NIH) called for the intentional incorporation of women in research studies; however, Black women were not explicitly considered until the 1993 NIH Revitalization Act (9), leaving them prone to further unexplored health disparities.

The recruitment of Black women in research can be further complicated if the research questions are focused on highly stigmatized or taboo topics. This study centers the responses of Black women who sought infertility treatment. Infertility is defined as the inability to conceive after 12 months of unprotected intercourse in women under 35 years, or after 6 months in women aged 35 years and older (10). Black women are known to experience infertility at rates as high as twice as much as non-Hispanic White women; however, they are two times less likely to seek treatment (11, 12, 13, 14). Part of these disparities in treatment utilization is due to the shame, guilt, and isolation surrounding infertility within the Black community (15). Further, scholarship in the realm of reproductive endocrinology has centered the voices and experiences of highly educated, affluent, and married non-Hispanic White women (12, 15, 16, 17, 18). Strategies for effective recruitment of Black women represent a large gap to explore, specifically in infertility, because of the negative psychological, financial, and emotional repercussions of infertility. The goal of this article is to explore factors influencing Black women experiencing infertility to willingly engage in a research study, with the aim of informing ethically responsive recruitment strategies.

The literature has documented many approaches to enhancing minority enrollment in medical research, such as snowball sampling, partnerships with community groups, and the use of media and social marketing techniques for targeted sampling (9). However, there are limited studies that form these intervention recommendations directly from the voices of the underrepresented groups. It is crucial that we address and eliminate the chasm between Black people and clinical research involvement, because those seeking healthcare in the United States become increasingly diverse, whereas inclusion of groups of color and women in clinical research has decreased over time (19, 20). The purpose of this study is to understand the factors that influenced a group of Black women to participate in a research study focused on infertility.

## Methods

Institutional review board approval was obtained at Duke University Health System and through the approval of the private infertility clinic’s research office.

### Study design and setting

We conducted a secondary analysis of semistructured interview data to understand factors that influenced Black women to participate in a research study. The parent mixed-methods study has been previously published (21). The parent study was a larger mixed methods study that combined retrospective chart review and semistructured interviews to examine factors associated with clinical decision making among a cohort of Black women who sought treatment at a large infertility clinic in the metropolitan Washington, D.C. area. To be included in the parent study, participants needed to be women who self-identified as Black/Black, between the ages of 18 and 44 years old, who presented to the clinic for an initial infertility evaluation with a male partner between January 2015 and September 2019. All interviews were conducted in English and lasted between 30 and 60 minutes. After the conclusion of the semistructured interviews, participants were asked a final open-ended question about what influenced them to participate in the larger study. We used data collected from this final question for this study. Participants self-identified as African American and/or Black; for consistency with current literature, the term ‟Black women” is used throughout this manuscript.

### Data collection & procedure

Purposive sampling was used to identify the qualitative sample. Research participants were identified from the clinic’s database. In addition, participants reached out to the principal investigator (PI) [M.C.] after seeing an advertisement on the clinic’s website or social media account, and additional participants were recruited through community events from September 2019 to January 2020. Regardless of the method of recruitment, participants were given the study’s recruitment flyer, a study information sheet, and a verbal consent form. Participants were given the option to complete the study in person or over the phone. For the qualitative and quantitative portions of the parent study, a total of 3,326 eligible patients were identified through the clinic database; 13 consented to participate in the qualitative portion, and 13 provided responses to the recruitment-related open-ended question included in this analysis.

All interviews were conducted by the PI over a medium of the participants’ choice (e.g., in person, over the phone). Participants were asked to complete a short demographic survey before the interview that collected sociodemographic data such as age, length of time experiencing infertility before seeking treatment, religious preferences, marital status, and level of education. The demographic survey was investigator-developed on the basis of prior infertility and health services research and reviewed by content experts for face validity before use. After the completion of the larger study’s interview question related to infertility treatment decision making, participants were asked to respond to an optional final question: Why did you choose to participate in this study? Patients received a cash incentive of $35 on the completion of the survey and the interview. Responses were recorded with an encrypted recording device. Interview audio was transcribed verbatim, and transcripts were checked against the audio recording twice for accuracy. Once by the first investigator and once by a paid research assistant.

### The interviewer

The interviewer who conducted all data collection did so as part of a nursing-focused dissertation study for a Doctor of Philosophy (Ph.D.) program. The interviewer is a US citizen by birth who identifies as a Black woman with Afro-Caribbean descent. At the time of the study, the interviewer was single, unmarried, had no children, was not actively attempting to conceive, and had not sought out or received any formal diagnosis for infertility. The study’s flyer included a picture of the interviewer along with pertinent contact information (email and personal cell phone number), the contact information for the interviewer’s research supervisors, and the contact information of the university's institutional review board. The interviewer had no prior affiliation with the fertility clinic where recruitment took place, as a researcher or as a patient. At the time of the study, the interviewer was funded by the Robert Wood Johnson Foundation Future of Nursing Scholars Program and the National Institutes of Health’s National Institute of Nursing Research as a predoctoral fellow. She was given access to the data and was supervised by the clinical research staff at the infertility clinic’s main office.

### Data analysis

Descriptive statistics were calculated using SAS v.9.4 to characterize the sample and are presented in Table 1. Final qualitative transcript data were uploaded into and analyzed with Nvivo v12. Each investigator used inductive thematic analysis to code data. Because this project was exploratory in nature, the use of inductive thematic analysis was appropriate for this study design (22). Inductive thematic analysis is a data-driven approach where themes emerge from the participants’ words and not through codes developed a priori (23). We followed the six-phase approach to thematic analysis as outlined by Maguire & Delahunt (24). All investigators were provided with transcripts and began familiarizing themselves with the data separately. Then, all of the investigators met to discuss initial codes and themes to sort out any discrepancies, agreements, or new insights. Final themes and subthemes were defined among the group, and all and investigators contributed to the final written report.

**Table 1:** Sociodemographic characteristics and recruitment types of interview participants (N = 13).

No.	Name^a^	Age	Income	Education^b^	Diagnosis	Method of recruitment^c^
1	Beyonce	36	>$100 k	G/P	Secondary infertility, unexplained	Clinic email
2	Meg	37	>$100 k	Some college	Bilateral tubal removal	Clinic website
3	Joy	43	>$100 k	College	Diminished ovarian reserve	Clinic email
4	Kathleen	34	>$100 k	G/P	Recurrent miscarriages, unexplained	Clinic email
5	Sylvia	39	>$100 k	G/P	Male factor	Clinic email
6	Jerry	42	>$100 k	G/P	Male factor	Community event
7	CeCe	43	>$100 k	G/P	Diminished ovarian reserve	Community event
8	Kiah	39	>$100 k	G/P	Secondary infertility; unexplained	Clinic email
9	Hannah	44	>$100 k	G/P	Unexplained infertility	Social media recruitment
10	Mary	32	<$99 k	G/P	Unexplained infertility	Clinic email
11	Ella	36	>$100 k	G/P	Diminished ovarian reserve	Clinic website
12	Geechie	40	<$99 k	G/P	Fibroids	Clinic website
13	Nae	39	<$99 k	High school	Unexplained infertility	Clinic website

Note:.

a All names presented are pseudonyms chosen by the participant or interview.

b Education reporting highest education level completed.

c Method of recruitment: Clinic email: participant was emailed by the principal investigator using the email provided to the clinic at their visit; Clinic website: participants contacted the principal investigator on the basis of the posting for the study on the clinic’s website; Community event: participant recruited from an in-person community event. G/P = graduate/professional degree.

## Results

### Sample characteristics

All 13 women who were interviewed in the parent study agreed to answer the question regarding factors influencing participation in the research project. Therefore, the final sample includes the responses of 13 Black women. The characteristics of the sample can be found in Table 1. Their ages ranged from 32 to 44 years old; most participants were legally married (n = 11), highly educated with at least a graduate level degree (n = 11), and all worked full time (n = 13). Interviews lasted between 30 and 80 minutes.

A total of three main themes were identified: Safety, Voice, and Altruism. Each of the themes came with a number of subthemes, which are all described below.

### Theme 1: Safety

Safety was referenced by 11 of 13 participants, with psychological and racial comfort most frequently emphasized. Safety was a recurring theme that included three subthemes of physical safety, psychological/emotional safety, and institutional trust.

It felt like it was harmless

On the point of physical safety, participants described the study as ‟harmless” and noted that no travel was needed to participate, the convenience of participating, and the lack of invasive procedures associated with the study. As two participants commented:

I'm not testing any medication; I'm not doing that. But some things, you know, that have been to help someone with their research, that's easy for me. A survey is easy…

But for your study, I just called you because it felt like it was harmless. It’s not like you were taking blood, just questions.

This person will be able to understand my story

In addition, psychological/emotional safety, particularly relating to racial comfort, was an important factor in participants’ perceptions of the research that ultimately influenced their participation. The interviewer’s/researcher’s race, gender, and appearance lent validity to the study. One participant stated:

I did see your picture. I was like, that lends some credibility to this survey. It feels like I will be heard. And that, like, this person will be able to understand my story.

Some participants were unsure if they would have participated if the interviewer had not been a Black woman:

Would I have not done it if it wasn't a black woman? Well, first of all, it would have to be a woman. I probably wouldn't have done it if it was a guy. But just comfort level but I probably would have done it. But I think I was quicker to say yes.

Although racial comfort was important for subjects’ feelings of psychological/emotional safety, the participants also noted the importance of the interviewer’s racial comfort in discussing medical issues. Participants commented that, in their experiences with the health care system, medical providers who were not Black struggled to discuss race:

I'll get to like say this, which I don't get to say because I didn’t say any of this to my nurses or my doctors. Like, you know what I mean, like, I didn't have, like, real conversations around my race. And how I feel like that might have played a part. You know, you don't want anybody to feel bad and like people jump to ‘Oh, you think I’m racist’ and it has nothing to do with it.

Another participant applied the same principle to researchers:

This is assuming that there would probably be a different experience or a different level of sensitivity because in others’ studies, I have to mention race and they’re not Black or in any way. They seem more uncomfortable even asking the questions. So, it does make a difference.

Institutional trust and verification

The final subtheme of safety was institutional trust and verification. Many participants mentioned that the academic institution and the foundation supporting the researcher were important factors affecting the perceived trustworthiness of the study. Further, many participants independently verified the researcher’s identity before responding to the recruitment flyer:

I think at the bottom you have something, some type of fellow or…? I forgot…Yeah. Yeah. [I] looked up the Robert Johnson Foundation on Google.

I did Google you to make sure you were real.

### Theme 2: Voice

All 13 participants identified the ability to have their voices and opinions heard as an important factor that contributed to their decision to participate in this study. This included three subthemes of self-expression, advocacy, and an opportunity to reveal the clinic’s practices.

I felt like my voice mattered

Participating in the study offered an opportunity for self-expression by reflecting and sharing their experiences as a Black woman with infertility. The women exuded a level of known confidence that involving their voices in the study was important and needed. As one participant stated:

I thought that I could have, I had something to add. I thought I felt like my voice mattered.

Whatever I have to say, it may be a pattern

The opportunity to give voice through advocacy for other Black women with infertility was also a strong influence in the study. Women discussed believing their involvement would give a voice to other women in similar circumstances. One participant noted:

I was like you know, whatever I have to say, it may be a pattern, it may be something that emerges from having multiple people, you know, who identify like me to say their experience(s).

When I saw the [clinic name]…I was like, okay

Participants also expressed their frustrations related to the experiences they had at this particular clinic, and stated that the opportunity to share their stories surrounding the care they received there motivated participation in the study. On the basis of the stories that some of the women shared, the care received by many participants felt ‟cookie-cutter” and not personalized:

So, the fact that you choose ∗clinic name∗ in particular, that was like, wow. There are probably other people who are experiencing the type of things, that, you know, I'm experiencing with that particular practice. And in speaking with my friend, the one who is a nurse and the one who’s an OBGYN, they both knew of ∗clinic name∗ in its early days before it became like this, industrial complex that it has become…They both, you know, have positive things to say about the doctors and practice. And even the practices here that became ∗clinic name∗, where it joined ∗clinic name∗ about what it was before and what it became after when they had begun practicing like a cookie cutter form of medicine. So when I saw the ∗clinic name∗ part in there, that's probably the thing that I was like, okay, if I have time to do it, then I will definitely do it.

Participation in the study provided both an opportunity to advocate for other women at that particular clinic and to share and process their own negative clinic experiences, which one participant said she hoped the clinic would read about:

I want [clinic name] to know the results [of this study] for real.

### Theme 3: Altruism

Ten of the 13 participants in the study felt strongly that it was important to provide support for the study. Specifically, they wanted their participation to help others. This ‟helping of others”, or altruism, spanned three subthemes: helping the researcher; helping to generate new knowledge; and helping other women.

Tryna help a Sista out

Participants in the study centered themselves on helping the first investigator, a self-identified Black woman with whom they identified with. They were invested in making sure they could help her in the completion of her dissertation project as described in the consent forms and recruitment flyer.

Well, I saw a black face and that and the fact that you were interested in researching something pertaining to Black women and I think that there will be great reward for all the work that you’re doing. So, when I saw it and I went through the description and I responded very quickly that of course I would help you.

You just seemed pleasant and doing good things. And you tryna do your thesis, so you know, tryna help a sista out.

Helping to generate new knowledge

The participants were eager to have their information be used to generate new knowledge for infertility access and decision making. They acknowledged that there was not enough information about infertility and Black woman and wanted to do their part to help generate important infertility information:

I don’t get to see a lot of women who look like me, but, you know, happy to participate because as I shared with you through this conversation, was that like the education is not out there. And, you know, however I may do my part.

For me, I am very passionate about educating and if I can do anything to put somebody else in a better position moving forward, then I’m more than happy to do that.

Oh, this is a sister trying to get some information about an issue that we don't have enough information about… Because I knew you were trying to work on information that may not exist yet. So yeah. So yeah, thank you, appreciate that.

Helping out other Black and Brown women with infertility

Beyond enhancing the scientific literature, participants were keen to help other women of color experiencing infertility within and beyond their communities. They felt that it was their duty and expectation that their contributions to the research would help other women of color receive appropriate infertility education, access, and treatment.

For me, I am very passionate about educating and if I can do anything to put somebody else in a better position moving forward, then I’m more than happy to do that. Because if that lady would have broken down to me just a little bit more, I would have maybe taken it a step further sooner to maybe find out, oh it’s not gonna be that easy for you based on these things when you decide to try to conceive and you may want to consider these options now.

## Discussion

When considering participating in clinical research, this sample of Black female participants reported that they participated in the research because they felt a sense of safety, believed they provided a valuable voice to the study, and felt an altruistic commitment to benefit more individuals beyond themselves. To our knowledge, this is the first study to report factors that influenced a group of Black women experiencing infertility to participate in research. Although a few other qualitative studies have explored the experiences of Black women in clinical research and found similar findings to ours, our study provides new additional insights for researchers to consider.

The study confirms previous work that highlights the importance of racially concordant participants and research staff members. As detailed in the study that explored Black women’s decision to participate in a focus group regarding breast cancer, participants voiced that the level of comfort participants have with those who look like them should not be undervalued or disregarded (21). However, our study provided new insights by highlighting the role of gender concordance. Participants in this study were positively influenced to participate because the interviewer was not only Black but also a woman. This could be possibly due to the nature of the topic centering around a woman’s health issue. However, this association of gender concordance with research participation should be further explored along with the interaction of gender and race. Further, the importance of relationship building to ensure a trusting relationship through genuine communication and culturally relevant issues has been an essential factor previously reported and still relevant to this study (9, 20).

One thing to note is that this group experienced a highly autonomous and independent decision-making process, which may relate to the isolation and stigma experienced by the participants with relation to their infertility. They were not pressured by family and friend circles to participate in the study, as other groups with stigmatized diseases may experience (20). Nevertheless, women did find it as an opportunity for self-reflection and felt like they understood the outcomes of participation, factors that were similarly relevant in other studies (20, 21). Finally, the appeal for the $35 cash incentive for participation was not mentioned as a motivating factor by participants, as it has been noted in other studies (20, 22). This could be due to the higher socio-economic status of this sample. All but one of the participants in this sample had a combined family income of more than $100,000 annually. The relative socioeconomic privilege of this sample likely shaped participants’ perceptions of research safety, autonomy, and altruism. Financial stability and health literacy may reduce perceived risk, increase confidence in navigating institutions, and support independent decision-making processes. These findings may therefore reflect how safety and trust are constructed among Black women with greater access to social and economic capital, rather than representing universal experiences across all Black women experiencing infertility. Finally, the desire to highlight the recruitment clinic’s practices was very important to the participants of this study; however, similar findings have not been discussed in other research examining recruitment strategies and would be worthy to explore.

### Recommendations

On the basis of the findings of this study, we can suggest preliminary recommendations to researchers who want to successfully recruit Black women into their studies. First, researchers must provide a sense of safety to the participants through multiple avenues. Limiting travel to and from research sites, providing flexibility in how data are collected, and providing times for data collection that are convenient for the participants would be ideal. Further, if studies require that more invasive procedures be done, providing opportunities to meet 1:1 with research staff to establish trust would be crucial for successful recruitment and education on study aims. Emphasizing psychological and emotional safety is necessary. Having trained researchers to deal with difficult conversations is also crucially important to help participants feel comfortable divulging their opinions and experiences on certain topics, especially if they are considered taboo or highly stigmatized.

Second, having racially and gender concordant principal investigators involved in early phases of research design and development is crucial in developing research aims and processes that are directly relatable to the targeted communities. Further, having these researchers’ actual faces, credentials, and appropriate level of contact information readily available could also enhance recruitment. Although only a subset of participants was recruited via digital platforms, many described independently verifying researcher identity online, suggesting that digital presence may play a supporting role in perceived legitimacy.

Finally, having a clear description of how valuable the participant is for the larger research would be important to emphasize. Not only did participants value the way they could contribute to the literature, but they also wanted to know and see how their research would be of service to others in similar communities and circumstances to theirs. Understanding these factors provided a source of inspiration and motivation to lend their time and voice to the project.

### Future research

Future research is needed to further elucidate effective and pragmatic strategies to be implemented in recruitment and data collection of communities of color. Research on the association of gender concordance should also be explored. There are also opportunities to further elucidate the effect of the intersectionality of these social identities and whether the interaction has a stronger influence than racial concordance alone. Intersectionality theory posits that overlapping social identities such as race and gender interact to shape lived experiences within systems of power (23). This study also highlights a great need for exploratory and interventional studies that seek to discuss and enhance researchers' comfort and proficiency with racially-influenced topics should also be explored. Finally, institutions should aim to enhance active recruitment and retention of researchers who come from diverse backgrounds and traditions to meet the needs of those who may be neglected by current scholars.

### Limitations

Despite high levels of rigor in data collection and data analysis, there were several limitations to this study design. To note, the sample was purposely recruited and therefore only included heterosexual, cisgender, highly educated, high-income, and partnered Black women of childbearing age. Consequently, the findings may not be generalizable to other groups of Black communities in the United States. Also, because this secondary analysis relied on a single open-ended question at a single time period rather than interviews designed specifically to explore research participation, certain dimensions such as evolving trust over time, nuanced concerns about institutional power, or contrasting perspectives from those who declined participation may not have been fully captured. Further, the question on recruitment was not the primary aim of the larger study design; therefore, recruitment until saturation of this research question was not considered. Saturation was on the basis of the larger study design, therefore, there could be additional factors that were not uncovered by this sample. Nevertheless, the findings provide a good foundation for future research focused on enhancing participation in research, especially on women’s health issues affecting women of color. Finally, we could not compare these responses with those who decided not to participate in the study. Understanding the process of decision making would allow us to understand what factors in the recruitment strategy possibly led eligible participants to decline participation.

## CONCLUSION

The findings of our study show that the process of decision making to participate in research among Black women is multidimensional and nonlinear. It dispels the myth that Black women are resistant to research solely on the basis of past racism in clinical research. Researchers should consider designing studies that promote multiple avenues of safety while allowing participants to feel they are a valuable piece of the research aims and outcomes.

## Declaration of Interests

M.C. has nothing to disclose. S.A. has nothing to disclose. R.O. has nothing to disclose. L.F. has nothing to disclose. L.L. has nothing to disclose.

## References

[1] Alarćon Garavito G.A., Gilchrist K., Ciurtin C., Khanna S., Chambers P., McNally N. (2025). Enablers and barriers of clinical trial participation in adult patients from minority ethnic groups: a systematic review. Trials.

[2] Alegria M., Sud S., Steinberg B.E., Gai N., Siddiqui A. (2021). Reporting of participant race, sex, and socioeconomic status in randomized clinical trials in general medical journals, 2015 vs 2019. J Am Med Assoc Netw Open.

[3] Fisher-Hoch S.P., Below J.E., North K.E., McCormick J.B. (2023). Challenges and strategies for recruitment of minorities to clinical research and trials. J Clin Transl Sci.

[4] Le D., Ozbeki H., Salazar S., Berl M., Turner M.M., Price O.A. (2022). Improving African American women's engagement in clinical research: a systematic review of barriers to participation in clinical trials. J Natl Med Assoc.

[5] Kaplan D.A. (2024).

[6] Cueva K.L., Arise A.R., Snyder C.R., Young B.A., Brown C.E. (2024). Medical mistrust among black patients with serious illness: a mixed methods study. J Gen Intern Med.

[7] Gary F.A., Thiese S., Hopps M., Hassan M., Still C.H., Brooks L.M. (2021). Medical mistrust among black women in America. J Natl Black Nurses Assoc.

[8] Hall O.T., Jordan A., Teater J., Dixon-Shambley K., McKiever M.E., Baek M. (2022). Experiences of racial discrimination in the medical setting and associations with medical mistrust and expectations of care among black patients seeking addiction treatment. J Subst Abuse Treat.

[9] Wieland M.L., Njeru J.W., Alahdab F., Doubeni C.A., Sia I.G. (2021). Community-engaged approaches for minority recruitment into clinical research: a scoping review of the literature. Mayo Clin Proc.

[10] American Society of Reproductive Medicine (2021). Infertility. https://www.asrm.org/topics/topics-index/infertility/.

[11] Cebert-Gaitors M., Shannon-Baker P.A., Silva S.G., Hart R.E., Jahandideh S., Gonzalez-Guarda R. (2022). Psychobiological, clinical, and sociocultural factors that influence Black women seeking treatment for infertility: a mixed-methods study. F S Rep.

[12] Dongarwar D., Mercado-Evans V., Adu-Gyamfi S., Laracuente M.-L., Salihu H.M. (2022). Racial/ethnic disparities in infertility treatment utilization in the US, 2011-2019. Syst Biol Reprod Med.

[13] Galic I., Negris O., Warren C., Brown D., Bozen A., Jain T. (2021). Disparities in access to fertility care: who’s in and who’s out. F S Rep.

[14] Richard-Davis G., Clarke L., Harris B., Jellerette-Nolan T., Thomas M. Caring for black women seeking fertility treatment: challenges, stigma, and hope. https://www.asrm.org/news-and-publications/news-and-research/announcements/caring-for-black-women-seeking-fertility-treatment-challenges-stigma-and-hope/.

[15] Weiss M.S., Marsh E.E. (2023). Navigating unequal paths: racial disparities in the infertility journey. Obstet Gynecol.

[16] Cebert M., Gonzalez-Guarda R., Stevenson E. (2021). Growing on (in) fertile ground: an evolutionary concept analysis of Black female fertility. Hum Fertil (Camb).

[17] Jackson-Bey T., Morris J., Jasper E., Edwards D.R.V., Thorton K., Richard-Davis G. (2021). Systematic review of racial and ethnic disparities in reproductive endocrinology and infertility: where do we stand today?. F S Rev.

[18] Barry D., Steinberg J.R., Towner M., Barber E.L., Simon M.A., Roque D.R. (2023). Enrollment of racial and ethnic minoritized groups in gynecologic oncology clinical trials: a review of the scope of the problem, contributing factors, and strategies to improve inclusion. Clin Obstet Gynecol.

[19] Pittell H., Calip G.S., Pierre A., Ryals C.A., Altomare I., Royce T.J. (2023). Racial and ethnic inequalities in US oncology clinical trial participation from 2017 to 2022. J Am Med Assoc Netw Open.

[20] Canidate S.S., Cook C.L., Varma D., Carnaby G.D., Ennis N., Stetten N.E. (2020). Recruitment, experience, and retention among women with HIV and hazardous drinking participating in a clinical trial. BMC Public Health.

[21] Frierson G.M., Pinto B.M., Denman D.C., Leon P.A., Jaffe A.D. (2019). Bridging the gap: racial concordance as a strategy to increase African American participation in breast cancer research. J Health Psychol.

[22] Chamie G., Kwarisiima D., Ndyabakira A., Marson K., Camlin C.S., Havlir D.V. (2021). Financial incentives and deposit contracts to promote HIV retesting in Uganda: a randomized trial. PLoS Med.

[23] Cho S., Crenshaw K.W., McCall L. (2013). Toward a field of intersectionality studies: theory, applications, and praxis. Signs.

[24] Maguire M., Delahunt B. (2017). Doing a thematic analysis: a practical, step-by-step guide for learning and teaching scholars. AISHE J.

